# tRNA-Derived Fragments (tRFs): Emerging New Roles for an Ancient RNA in the Regulation of Gene Expression

**DOI:** 10.3390/life5041638

**Published:** 2015-11-27

**Authors:** Simon P. Keam, Gyorgy Hutvagner

**Affiliations:** Centre for Health Technologies, Faculty of Engineering and Information Technology, University of Technology Sydney, Ultimo 2007, Australia

**Keywords:** tRNA-derived fragments, tRF, tRNA, small RNA, gene regulation

## Abstract

This review will summarise the recent discoveries and current state of research on short noncoding RNAs derived from tRNAs—known as tRNA-derived fragments (tRFs). It will describe the features of the known subtypes of these RNAs; including sequence characteristics, protein interactors, expression characteristics, biogenesis, and similarity to canonical miRNA pathways. Also their role in regulating gene expression; including mediating translational suppression, will be discussed. We also highlight their potential use as biomarkers, functions in gene regulation and links to disease. Finally, this review will speculate as to the origin and rationale for the conservation of this novel class of noncoding RNAs amongst both prokaryotes and eukaryotes.

## 1. Introduction

The recent increase in rapid and inexpensive RNA sequencing has led to the discovery of a myriad of novel noncoding RNA species. These studies have also shed light on the variations in small RNA expression amongst different organisms, developmental stages and disease states (reviewed in [1]). This has resulted in a rich catalogue of RNAs with biological functions that includes well-established players such as endogenous siRNAs (endosiRNAs), microRNAs (miRNAs) and PIWI-interacting RNAs (piRNAs).

There is mounting evidence that >85% of the human genome is readily transcribed [2], and it has become clear that regulatory small RNAs can be processed from novel RNA sources (reviewed in [3,4]). However, the functionality of small RNAs from abundant species of “housekeeping” noncoding RNAs (e.g., rRNA, tRNA, snRNA, snoRNA, *etc.*) remains a highly studied topic. In this review, we examine the current state of research on short RNAs derived from transfer RNA (tRNA); called tRNA-derived fragments (tRFs).

## 2. Characteristics and Biogenesis of tRFs

**Figure 1:**
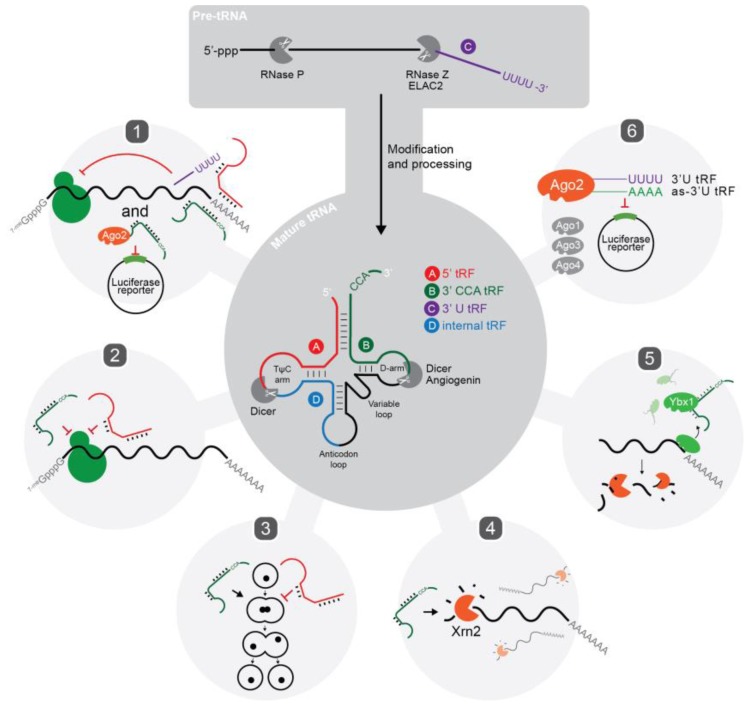
tRNA biogenesis, types of tRFs, nucleases involved in their biogenesis, and examples of their functions. Outline of mature tRNA biogenesis (dark grey) from pol(III)-transcribed pre-tRNA precursors which undergo nuclease cleavage by RNase P and RNase Z, enzymatic modification including addition of CCA trinucleotide and folding into mature tRNA. Four recognised types of tRFs and their identified nucleases (if any) shown including (A) 5′ tRF; (B) 3′ CCA tRF; (C) 3′ U tRF; (D) itRF. Examples of functions for tRFs in the literature (light grey) including: (**1**) gene repression of mRNAs via 3′UTR targeting [9,10] and reporter construct silencing [11]; (**2**) targeting of ribosomes and inhibition of translation [12,13]; (**3**) modulators of cell proliferation [14]; (**4**) promotion of Xrn2-mediated exonuclease degradation of mRNAs [15]; (**5**) displacement of Ybx1 protein from mRNA and promotion of degradation [16]; (**6**) sense-induced trans-silencing [17].

Transfer RNAs are an extremely conserved and highly abundant RNA species with a well-defined role in protein translation. The biogenesis of mature tRNAs (reviewed in [5] and shown in Figure 1) results from the transcription of pre-tRNA and subsequent trimming of the 5′ and 3′ ends by the endonucleases RNase P and RNase Z, respectively. In addition, introns are present in a small percentage of eukaryotic tRNAs and are spliced out by tRNA-specific enzymatic complexes [6]. Following extensive post-transcriptional modification and folding, mature tRNAs are exported to the cytoplasm. A properly folded tRNA contains four distinct arms: the D arm, anticodon loop, TψC arm and variable loop (see Figure 1). A 3′ CCA trinucleotide is also added at the acceptor stem by a nucleotidyl transferase as it is not genomically encoded [7]; with the exception of certain bacterial species, including *Escherichia coli*, that do encode it [8].

### 2.1. tRNA-Halves versus tRNA-Derived Fragments (tRFs)

tRNA-derived short RNAs can be broadly classified into two main groups; tRNA-halves and tRNA-derived fragments; likely depending on their biogenesis. Half-tRNAs are produced by ribonucleolytic cleavage of mature tRNAs under stress conditions by Angiogenin in higher eukaryotes [18,19] and by the RNase Rny1 in yeast [20]. In eukaryotes, these have been referred to as tRNA-derived stress-induced small RNAs (tiRNAs) [19] and correspond to a single cleavage of mature tRNA in the anticodon loop. However, there is ambiguity over whether some tRNA-derived short RNAs are classified as tRNA-halves or tRFs in many transcriptome studies. This is due to the absence of stress conditions which would typically favour the production of conventional half-tRNAs or tiRNAs. In contrast, transfer RNA-derived RNA fragments (tRFs) are a seemingly distinct group of small RNAs generated through the endonucleolytic cleavage of both mature and precursor tRNAs, usually near the D- or TψC-arm [20,21]. In this review, we mainly consider these tRFs that are not derived from a sole cleavage site in the anticodon loop and are shorter than 32 nt. Also considered will be long (>35 nt) tRFs that do not involve canonical anticodon cleavage. A summary of tRFs identified in the literature is shown in Table 1.

**Table 1:** Types of tRFs identified in the literature.

Group	Organism/ Cell Type/Cell Line	Length (nt)	Type(s)	Nuclease-Identified	Co-Factor(s)	Ref.
Plant	Phosphate-starved *A. thaliana* roots	19	5′			[22]
	Rice (*Oryza sativa*) embryogenic callus	N.S	5′ and 3′			[23]
	*Brassica rapa*	27–30, 15–18, 23	5′			[24]
	*Arabidopsis thaliana*	19	5′ and 3′ CCA		Ago1,2,4,7	[25]
	Barley (*Hordeum vulgare* L) Normal and phosphate-starved conditions	N.S	N.S			[26]
Archaea	*Haloferax volcanii*	40	3′ U			[27]
	*Haloferax volcanii*	26	5′		Small ribosomal subunit	[12]
Fungi	*Magnaporthe oryzae*	~35	Mainly 5′			[28]
	*Phytophthora infestans*	25–30 (peaks at 27 and 30)	5′	Dcl1-independent; Ago1 and 4 dependent		[29]
Flatworm	*Schistosoma japonicum* eggs	19–25	5′			[30]
Protozoa	*Tetrahymena*	~23	3′ CCA		Twi12	[31]
	*Tetrahymena*	18–22	3′ CCA		Twi12	[15]
	*Giardia lamblia*	18–32	5′ and 3′			[32]
	*Trypanosoma cruzi*	N.S	N.S			[33]
	Exosomes from *Leishmania donovani*, *Leishmania braziliensis*	38–46	5′ and 3′ CCA			[34]
Yeast	*Schizosaccharomyces pombe*	23	5′	Dicer-independent		[35]
Insect	*Drosophila* (multiple species)	23–29	5′ and 3′ CCA		Ago1 and Ago2	[36]
	*Bombyx mori*	5′: 33 and 183′ CCA:40 and 21	5′ and 3′ CCA		BmAgo2	[37]
Mammal	KSHV-infected primary-effusion lymphoma cell line; MEFs	5′: 14–15 3′ CCA: 17–18	5′ and 3 CCA'	Dicer/DGCR8-independent	3′ CCA/Ago2 in MEF	[38]
	5-8F (nasopharyngeal carcinoma)	19	3′ U			[39]
	Mature human B-cells	22	3‘ CCA	Dicer	Ago1–4	[10]
	HEK293 (kidney) and HCT116 (colon carcinoma)	20–22	3′ CCA, 3′ U	Dicer (3′ CCA) RNase Z (3′U)	Ago3/4 > Ago1/2	[11]
	Mouse embryonic stem cells	21	3′U	Dicer		[40]
	HIV-1 infected MT4 cells	18	3′ CCA	Dicer	Ago2	[17]
	LNCaP and C4-2 (prostate carcinoma)	18–22	5′, 3′ CCA and 3′ U	Rnase Z for 3′U		[14]
	HeLa (cervical carcinoma)	19	5′	Dicer	Ago1/2 (poorly)	[41]
	HepG2 (liver carcinoma)	22	3′ CCA			[42]
	HeLa (cervical carcinoma)	19–21	5′		Polysomes	[13]
	MDAMB231 (human breast carcinoma)	27–29	5′		Piwi-l4	[43]
	Healthy and cancerous human tissue	19–30	5’, 3’and itRF			[44]
	Human breast cancer cell lines	59–87	All		Ybx1	[16]
	Human testis and testicular germ cell tumour	25–36	5′			[45]
	Exosomes from human semen	18–19, 30–34	5′			[46]
	Exosomes from human dendritic cells and T-cells	30, 40–50	3′ CCA			[47]
	*CLP1*-mutant mouse embryonic fibroblasts (MEFs)	41–46	5′ intronic			[48]
	Extracellular vesicles from MCF7/MCF10A (breast cancer)	18	3′			[49]
Multiple	Bacteria, yeast, nematode, fly, mouse and human tissues and cancer cell lines	5′: 15,22,32, 3′ CCA: 18–22	5′, 3′ CCA and 3′ U	Dicer/DGCR8-independent in mice, fly and *S.pombe*	Ago1, 3, 4 (5′ and 3′ only)	[50]

tRF: transfer RNA-derived fragments. N.S: Not specified.

### 2.2. Types of tRFs and Nomenclature

Studies reporting on the different types of tRFs are yet to retain a consistent nomenclature to describe them. In this review, we maintain the nomenclature we proposed previously [51]. Here, we classify four main types of tRF based on their position of origin in pre-tRNA or mature tRNA (see Figure 1). The 5′ tRFs are generated from a cleavage in the TψC-arm of mature tRNA extending to the 5′ end of the molecule. The 3′ tRFs are a second class of tRFs that originate from a cleavage in the D-arm and includes the trinucleotide CCA post-transcriptional modification. A third class of tRF is sourced from the 3′ end of a pre-tRNA molecule—known as 3′ U tRFs. Here, cleavage is typically performed by RNase Z and results in the presence of characteristic poly-U residues at the 3′ terminus. Another more recently characterised tRF are those derived from a combination of cleavages in the anticodon loop and either D-arm or TψC-arm, collectively termed internal tRFs (itRFs) [44].

### 2.3. Biogenesis of tRFs

The production of small RNAs from tRNA precursors is an emerging field of investigation. Aside from the production of half-tRNAs via the cleavage of mature tRNAs under stress conditions, little is known about the biogenesis of shorter (<32 nt) tRNA fragments. In some cases, tRFs appear to be produced in a manner similar to the canonical miRNA pathway. Therefore, focus has been put on identifying if components of the miRNA processing pathway (Dicer, Drosha, Dgcr8 *etc.*) are required for tRF biogenesis. Until recently, tRF production appeared to be Dicer-dependent in mammals [11,17,40,41], however subsequent analyses have shown that both 5′ and 3′ tRFs can be produced independently of Dicer and Dgcr8 in HEK293 cells [38]. Recent work has confirmed that the canonical miRNA machinery is dispensable for the production of tRFs in *Phytophthora infestans, Drosophila*, mice and *Schizosaccharomyces pombe* [29,35,50]. 

Mature tRNA biogenesis normally requires the endonuclease RNase Z to trim the 3′ trailer from pre-tRNA. In mammalian cells, this enzyme has been shown to be required for 3′U tRF generation in cultured human cells [11,14]. Another tRNA endonuclease, Elac2/RNaseZ^L^, has been demonstrated to be required for generation of a 3′U tRF named tRF-1001 in human colon cancer cells [14]. Angiogenin is also capable of producing short 3′ tRFs in *in vitro* cleavage assays by cleaving in the TψC arm [38]. Other types of tRNA-fragments can be produced by Angiogenin under non-stress conditions [20,52,53], and in mouse embryonic fibroblasts deficient in the tRNA kinase Clp1 [48].

The reason for hypervariability in mechanism of tRF generation is yet to be fully understood, particular whether it is a result of multiple mechanisms or other cell-specific factors that dictate the types of tRF produced. In addition, tRFs may be generated by the aberrant recognition and cleavage of misfolded tRNAs. The correct modification of tRNA lowers conformation flexibility and increases thermal stability (reviewed in [54]). A loss of this stability and associated increases in flexibility may cause tRNAs to become the substrate for various endonucleases. Nevertheless, is appears that tRFs can be produced independently of well-known endonucleases such as Dicer, leaving the possibility for yet unknown mechanisms to contribute to their production.

### 2.4. Proteins Associated with tRFs

Due to the increase in reporting on tRFs, much attention has been brought to identifying the proteins and pathways they interact with. Many studies have since identified binding partners which may provide clues to their possible functions. As many tRFs share similar features to RNAs in the siRNA and miRNA pathways, many groups have focused on identifying which (if any) of the Argonaute proteins associate with tRFs. Couvillion and colleagues were first to identify that Twi12, a Piwi/Ago protein homolog, is a binding partner of a 23 nt tRF in the ciliate protozoa *Tetrahymena thermophila* [15,31].

The first description of Argonaute/tRF interactions in more complex eukaryotes was reported by Haussecker *et al.* Here, both 3′ U and 3′ CCA tRFs displayed a preference for binding Ago3/4 over Ago1/2 in HEK293 cells [11]. Subsequent analyses have identified that both 5′ and 3′CCA tRFs bind Ago2 in mouse embryonic fibroblasts (MEFs) [38] and the human T-cell leukemia cell line (MT4) [17], respectively. Other analyses have shown that 5′ tRFs poorly associate with Argonaute proteins Ago1 and Ago2 [41] in the human cancer line HeLa. This finding has been largely verified in bioinformatics analyses of PAR-CLIP data from Ago1–4 immunoprecipitations from HEK293 cells, where both 5′ tRFs and 3′ CCA tRFs display binding affinity for Ago1,3 and 4 but not Ago2 [50]. 5′ tRFs have also been demonstrated to bind the human Piwi protein Hiwi2 in a breast cancer cell line [43]. Interestingly, tRFs also appear to be differentially loaded onto Ago proteins depending on minor differences in cell subtype [44]. Studies in less complex organisms support the notion that tRFs associate with Argonaute proteins in plants [25], silkworm [37] and fly [36].

The association of tRFs with the translational machinery has also been investigated. Two studies have shown that both 19 and 26 nt 5′ tRFs associate with polysomes in HeLa cells [13] and in the halophile *Haloferax volcanii* [12], respectively. Importantly, both these studies demonstrated that protein synthesis is down-regulated as a result of this binding.

### 2.5. Subcellular Localisation

A great deal of information about the function and biogenesis of small noncoding RNAs stems from the subcellular localization of their precursor and mature components. For example, the miRNA pathway comprises pri-miRNAs and Drosha-processed pre-miRNA precursors in the nucleus and Dicer-processed and Ago-loaded mature miRNAs in the cytoplasm. In contrast, the subcellular compartmentalisation of tRF precursors and biogenesis is much less understood. Limited evidence suggests that the vast majority of mature 5′ and 3′ CCA mammalian tRFs are cytoplasmic [11,39], with large proportions of the nuclear-processed 3′ U tRFs are also exported to the cytoplasm. Lee and colleagues have also shown that 3′ U tRFs can be processed by the endonuclease *Elac2* after export to the cytoplasm [14]. In contrast, Kumar and colleagues demonstrated that 5′ tRFs are more abundant in the nucleus of human HeLa cells [50]. Further analysis of diversity in the location and processing of tRFs may be indicative of separate functions within the cells. If tRFs are involved in gene regulation, the prevalence of cytosolic fragments is suggestive of translational and/or RNA control, rather than transcriptional or epigenetic regulation. In addition to the localisation of tRFs inside cells, numerous studies have reported on tRFs being expressed in extracellular vesicles and exosomes secreted from mammalian and protozoan cells. Here, 5′ and 3′ CCA tRFs have been identified in exosomes secreted from human semen [46] and human dendritic cells and T-cells [47], respectively. In addition, the intracellular parasites *Leishmani donovanii* and *Leishmania braziliensis* release exosomes containing long 5′ and 3′ CCA tRFs [34].

### 2.6. tRNA-Derived Fragments Are Deeply Conserved in Nature

A common theme that has been identified by deep sequencing surveys of small ncRNAs is the universality of tRFs in almost every branch of life. To date, tRFs of any of the three subtypes have been documented in bacteria [50], algae [29], archaea [12,27], protozoa [15,31,32,34], flatworms [30], plants [22,23,24,25,26], yeast [35,50] and mammals [11,13,14,16,17,23,38,39,40,41,42,43,44,45,50]. Also, the online database tRFdb reveals 552, 559, 433, 320 and 649 tRF sequences from human, mouse, *Drosophila*, *S.pombe* and *C. elegans*, respectively [55]. This is perhaps not surprising considering the universality of precursor tRNA throughout all taxonomic groups. However, tRFs are expressed in less complex organisms, such as bacteria and archaea, which do not possess the canonical miRNA or siRNA pathway. Numerous studies have shown that tRFs can be produced in the absence of many of these conventional ribonucleases, and therefore may be indicative of an underlying more ancient regulatory system that is not dependent on canonical gene silencing pathways. Importantly, even though prokaryotes lack much of the typical miRNA processing machinery, they do however universally express several isotype-specific tRNA endonucleases [56,57].

The conservation of these and other related endonucleases in eukaryotes suggests that tRF biogenesis may be a fundamental pathway overshadowed by more recently evolved and perhaps more specific regulatory pathways. A complicating aspect of this is the deep conservation of tRNA-half generation under oxidative and/or nutritional stress [19,58,59,60]. Distinguishing these two prevalent types of tRNA-derived small RNAs will be crucial to elucidate if they have mutually exclusive or shared functions. Studying tRFs poses an interesting experimental challenge both bioinformatically and biochemically, owing to the abundance of their precursor transcripts and frequency of contamination in NGS data. Strategies that overcome these problems center on correctly identifying the origin of tRFs, and are an emerging bioinformatic discipline [61,62].

## 3. Functionality and Significance of tRFs

Despite mounting evidence for the generation of tRFs in almost all cell types, a recurring concern is that tRFs are aberrant degradation products of endonuclease activity. A common response to this is typically three-fold. Firstly, numerous groups have demonstrated that the processing of mature tRNA into tRFs is remarkably site-specific, generating tRFs with highly defined lengths amongst different cell types. Secondly, tRF expression does not correlate with the abundance of their respective precursor tRNAs [22,41,43,44]; with the exemption of those identified in *Tetrahymena* [31]. The generation of tRFs appears to be restricted to specific isotypes of tRNAs, in some cases dictating which endonuclease is involved. This suggests that tRNA isotype selection and processing is non-random. Finally, tRFs exhibit features of functional regulatory molecules, some of which are shown in Figure 1.

### 3.1. miRNA-Like Regulation of Gene Expression

Until recently, a major deficiency in our understanding of tRF biology was that there was little known about the function of tRFs once they were generated. Perhaps the most powerful function tRFs could have would be the ability to behave like miRNAs and siRNAs and repress the expression of endogenous targets. Indeed, several studies have demonstrated the ability of both 5′ tRFs and 3′ CCA tRFs to target the 3′UTR of specific mRNAs and repress their translation. The first exhibition of this was a 22-nt long 3′ CCA tRF that uses canonical miRNA machinery to repress replication protein A1 (*RPA1*) mRNA, among other genes [10]. More recently, Deng and colleagues demonstrated that the 3′ portion of a 5’ tRF (named tRF5-GluCTC) targets the 3′UTR of *APOER2*. Remarkably it was shown that tRF5-GluCTC is overexpressed in response to respiratory syncytial virus (RSV) infection and promotes further infection by modulating the level of Apoer2 [9]. These reports are in addition to the numerous instances of annotated miRNAs with silencing capacity that are derived from tRNAs which is discussed in the next section.

Prior to this, similar forms of luciferase reporter construct silencing have been observed in the human cancer cells HEK293 and HCT116. Here, the inhibition of a 3′ CCA tRF (cand14) with an antisense oligonucleotide resulted in derepression of the reporter construct by ~30%–40%. In the same study, a 3′ U tRF (cand45) alone did not appear to repress its target. However unexpectedly, the addition of an antisense strand to cand45 resulted in a dramatic silencing of the luciferase target by ~80%. This mechanism was coined sense-induced *trans*-silencing (SITS) [11]. Some studies propose that tRFs may serve to bind complementary RNAs and form duplexes that the canonical miRNA machinery can recognise and cleave [17].

### 3.2. The tRF/miRNA Mixup

A consequence of the advent of advanced bioinformatic methodologies has been a rapid influx of novel noncoding small RNAs. Recent findings have suggested that there is misannotation and cross-mapping between miRNA precursors and other types of ncRNAs (e.g., tRNA, rRNA *etc.*). This phenomenon was identified *in silico* in 2010 and 2011 by identifying eight tRFs incorrectly annotated as miRNAs (miR-tRFs) [63,64]. Those specifically identified were hsa-miR-1280, hsa-miR-720, hsa-miR-1308 and hsa-miR-886-5p [63], plus hsa-miR-4284 and hsa-miR-3182 [64]. Both studies identified hsa-miR-1274a and hsa-miR-1274b [63,64]. Most of these have since been removed from miRBase, but interestingly, hsa-miR-4284 and hsa-miR-3182 remain at the time of preparing this manuscript. These two miRNAs have been independently identified in Ago2 immunoprecipitates from either human stem cells (hsa-miR-4284) [65] or melanoma (hsa-miR-3182) [66].

More recent studies have shown that many of these miR-tRFs exhibit differential expression in human tissues and cancer. It has been demonstrated that the oncoprotein YB-1 interacts with a suite of noncanonical small RNAs in breast cancer, including two miR-tRFs; miR-4284 and miR-1308 [63,64,67]. Human skin has also been shown to express two ~22 nt tRFs derived from tRNA^Ile(TAT)^ and tRNA^Pseudo(TTA)^ [68], the former of which is a homolog of a mouse miRNA (mmu-miR-1983). A miR-tRF derived from tRNA^Leu^ (annotated as hsa-miR-1280) has also shown to be elevated in human breast cancer tissue [69]. Enrichment of the miR-tRFs miR-720 (tRNA^Thr^) and miR-1274b (tRNA^Lys^) have also been observed in serum-deprived extracellular vesicles (EVs) secreted from human breast cancer cells [49].

Finally, one study has identified a 22 nt tRF named CU1276, which is derived from the 5′ end of the tRNA^Gly(GCC)^ and is down-regulated in lymphoma and primary cancers. This tRF associates with Ago proteins, is dependent on Dicer for biogenesis, and represses endogenous genes (e.g., *RPA1*) by targeting 3′ gUTRs [10]. This is a chief example of a tRF behaving like a miRNA but not being derived from a canonical pri-miRNA gene.

### 3.3. Regulating Translational Efficiency

Aside from a role for tRFs in mediating miRNA/siRNA-like silencing, several studies suggest a role for tRFs in global translational repression. In the archeabacteria *Haloferax volcanii,* Gebetsberger and colleagues showed that a 26-nt 5′ tRF (tRNA^Val^) directly binds the small ribosomal subunit and reduces translation by inhibiting peptidyl transferase activity. [12]. In human cells, a 19-nt 5′ tRF derived from glutamyl tRNA (Gln19) was able to repress a luciferase assay target irrespective of the presence of canonical miRNA-like target sites in HeLa cells. Further work showed that the mechanism is likely targeting translational elongation [13]. An interesting motif identified in human tRFs is a GG-dinucleotide at base-positions 17–18 in 5′ tRFs derived from tRNA^Gln^, tRNA^Glu^ and tRNA^Val^ [13]. Artificial tRFs containing this GG-dinucleotide motif but other random sequences maintained their function in translational repression.

### 3.4. tRFs Roles in the Regulation of Cell Viability, RNA Degradation and RNA Stability

Other putative roles for tRFs that have been identified include the regulation of proliferation and cell viability and modulating the turnover of different RNAs. Lee and colleagues have previously showed that (tRF-1001) is essential for cell proliferation, and results in G2 accumulation and reduced viability in HCT116 cells [14]. Interesting, this tRF-1001 is identical to cand45, previously reported by Haussecker and colleagues [11]. Another tRF that shows proliferative inhibition is the 5′ tRF named (CU1276). Here, proliferation is hypothesized to be suppressed via the downstream loss of Rpa1 functionality [10].

RNA stability is another key mediator of gene regulation, determining the rates at which RNA molecules are degraded by exonucleases; ultimately changing protein levels. Two reports have demonstrated that 3′ CCA tRFs can modulate at least two different RNA turnover mechanisms. In *Tetrahymena*, 3′ CCA tRFs have been shown to stimulate the activity of the nuclear 5′ to 3′ exonuclease Xrn2 [15]. In breast cancer cells, a subset of 3′ CCA tRFs contain putative binding sites for the RNA-binding protein Ybx1, which is known to stabilise oncogenic transcripts. Here, tRFs competitively bind Ybx1 and displace mRNA transcripts; reducing their stability and promoting degradation [16].

### 3.5. tRFs Association with Diseases; Potential Biomarkers

Half-tRNAs have a relatively well described role in disease and infection [9,33,61,70,71,72,73,74,75]. The first hints that tRFs are important in disease states was described in 2009. Here, an 18-nt long 3′ tRF was found to be dramatically upregulated in response to HIV infection in the human T-cell line MT4 [17]. Also, the human pathogen Giardia lambia induces expression of several tRFs during infection and differentiation, suggestive of possible modulation of host genes [32]. 

The available literature signifies that tRFs are predominately expressed in highly proliferative human cancer cells [11,13,14,16,17,38,39,41,42,43,44,45,50]. This could simply be a product of the popularity and power of human cancer cells as model organisms. However, many studies report an association between tRF expression and higher rates of proliferation [13,14]. 3′ CCA tRFs have been demonstrated to repress proliferation and the DNA damage response in B-cell lymphoma cells [10]. A short 10–16 nt tRF (of unknown type) has also been shown to repress growth in human urinary bladder carcinoma [76]. The overwhelming evidence suggests that the expression of tRFs and cell proliferation are deeply entwined; possibly suggestive of a use in medicine for the manipulation of highly proliferative cells such as cancer cells.

The heterogeneity and stability of some small RNAs makes them useful as biomarkers for diseases such as cancer. Telonis and colleagues have reported that the transcriptional abundance of different tRFs can distinguish normal breast tissue from triple negative (ER-/PR-/HER2-) and triple positive breast cancer ER+/PR+/HER2+ [44]. In other breast cancer studies, tRFs have been shown to regulate tumour-suppressive genes and are associated with less tumorigenic breast cancer cell types [16]. Finally, Rounge and colleagues identified three 5′ tRFs that are differentially expressed in testicular germ cell tumours (TGCTs) [45].

## 4. Rationale for Evolution of tRFs

Despite much research into cataloging the expression of tRFs, little progress has been made on identifying if they are bona fide regulators of genes or by-products of improper tRNA processing. This section aims to discuss possibilities for the expansion of this novel noncoding RNA.

### 4.1. tRFs: The Forgotten Gene Regulation Mechanism

tRFs are expressed in all of the three domains of life; eukarya, archea and bacteria. Bacteria do not express the canonical small RNA pathway (e.g., Dicer, miRNA, *etc.*), however they do appear to have their own rudimentary gene regulatory system that uses small RNAs (for review see [77]). The cleavage of tRNAs by nucleases into various small RNAs has also been observed in bacterial species [77,78]. It should be noted however that as yet, these tRFs have not been shown to exhibit gene regulatory functions. Nevertheless, we may therefore speculate that more complex organisms have retained such an ancient pathway. Furthermore, as tRF expression appears to be linked to diseases such as cancer, it may be that the tRF pathway is reactivated only upon the loss of RNA homeostasis in unhealthy cells. Interestingly, the expression level of highly defined tRF species seems to rival and in some cases exceeds that of miRNAs, especially in cancers [10,14,41]. Whether or not this increased expression correlates with additional function remains to be investigated. A possibility we propose is that tRFs are part of an ancient regulatory pathway that has been co-opted for use by more recently evolved counterparts.

### 4.2. tRFs: Function Following Fortune

tRF expression appears to be intricately linked with proliferation, especially in cancer cells. This may be because uncontrolled transcription leads to inadequate modification of mature tRNAs which hinders their ability to properly fold. The formation of hairpin structures could lead to recognition by endogenous nucleases such as Dicer and production of short RNAs [40]. This phenomena has already been observed for half-tRNAs [70]. The generation of tRFs may simply be a product of rampant transcription in cancer cells that drives the recognition of tRNAs by endonucleases.

A hallmark of some cancers is the disruption of the normal miRNA pathway (for review see [79]). Recent studies have reveal that Dicer is essential for the normal loading of miRNAs onto Ago2 protein and formation of competent RISC complexes [80]. Here, the loss of Dicer results in the formation of RISC complexes loaded with small RNAs from unconventional sources. As tRFs have been shown to associate with Ago proteins in virtually all animals, the possibility remains that the disruption of the miRNA pathway in cancer leads to the accumulation of tRFs bound to Ago protein; promoting their stability. However, the presence of tRFs in non-cancerous less complex organisms, and inconsistent reports for the requirement for Dicer-mediated biogenesis, suggests this pathway is not solely responsible for tRF generation. Interestingly, one report has demonstrated that whilst Ago2 binds tRFs in cancer cells, they cannot repress gene targets [81]. This poses the interesting possibility that tRFs may function by binding and sequestering free Ago proteins and preventing them from targeting mRNAs for silencing. This may help to explain why tRFs have been observed to have non-specific effects on translational efficiency. 

### 4.3. miRNA Sources Extend beyond pri-miRNA Genes

It has become increasingly clear that miRNAs are not always generated from hairpin pri-miRNA structures, and that many other sources are likely to be possible [82]. As previously mentioned, numerous miRNAs previously annotated on miRBase have been demonstrated to be derived from tRNAs. Interestingly, most are now regarded as not “true” miRNAs. Much of tRNA biogenesis occurs in the nucleus prior to cytoplasmic export, meaning the primary endonuclease enzymes responsible for miRNA biogenesis (Dicer and Drosha) may also generate miRNAs from mature tRNAs as part of normal gene silencing pathways. Is it plausible that tRFs evolved in parallel with miRNAs in higher eukaryotes and represent an alternative source of RNA as guides for gene regulation? The lack of mechanistic and functional data for most tRFs leaves this question open. Short RNAs have also been shown to be produced from a wide variety of other ncRNA species including snoRNAs, ribosomal RNAs, vault RNAs, etc [83]. It is therefore possible that tRFs represent a subset of noncanonical short RNAs that are similarly processed and have gained regulatory roles in conjunction with miRNAs. 

## 5. Concluding Remarks

Perhaps the most pertinent question is whether there is a defined function or established evolutionary pathway for tRFs. Research to date is still in the phase of identifying such RNAs and their functional studies are limited so far. Although some isolated functions have been indicated, the vast majority of tRFs appear to operate via unknown mechanisms. Identifying if these pathways are conserved between different species could unravel the biology of this new class of noncoding RNAs. Also, describing mechanistically how tRFs regulate translation would assist our understanding of their biology. This may resolve the hypothesis that tRFs act by binding and abrogating the miRNA pathway. Nevertheless, our current understanding of tRFs suggests they are not merely byproducts of random cleavage of tRNAs, yet remain as possible mediators of translational and/or gene regulation.
